# Insulin‐Stimulated Bone Blood Flow and Bone Biomechanical Properties Are Compromised in Obese, Type 2 Diabetic OLETF Rats

**DOI:** 10.1002/jbm4.10007

**Published:** 2017-06-12

**Authors:** Rebecca K Dirkes, Laura C Ortinau, R Scott Rector, T Dylan Olver, Pamela S Hinton

**Affiliations:** ^1^ Department of Nutrition and Exercise Physiology University of Missouri–Columbia Columbia MO USA; ^2^ Division of Gastroenterology and Hepatology Department of Medicine University of Missouri–Columbia Columbia MO USA; ^3^ Research Service Harry S Truman Memorial VA Hospital Columbia MO USA; ^4^ Department of Biomedical Sciences University of Missouri–Columbia Columbia MO USA

**Keywords:** ANALYSIS/QUANITIFICATION OF BONE, BONE QCT/µCT, ANALYSIS/QUANITIFICATION OF BONE, DXA, ANIMAL MODELS, GENETIC ANIMAL MODEL, ORTHOPAEDICS, BIOMECHANICS, DISEASES AND DISORDERS OF/RELATED TO BONE, OTHER

## Abstract

Type 2 diabetes (T2D) increases skeletal fragility and fracture risk; however, the underlying mechanisms remain to be identified. Impaired bone vascular function, in particular insulin‐stimulated vasodilation and blood flow is a potential, yet unexplored mechanism. The purpose of this study was to determine the effects of T2D on femoral biomechanical properties, trabecular microarchitecture, and insulin‐stimulated bone vasodilation by comparison of hyperphagic Otsuka Long‐Evans Tokushima Fatty (OLETF) rats with normoglycemic control OLETF rats. Four‐week old, male OLETF rats were randomized to two groups: type 2 diabetes (O‐T2D) or normoglycemic control (O‐CON). O‐T2D were allowed *ad libitum* access to a rodent chow diet and O‐CON underwent moderate caloric restriction (30% restriction relative to intake of O‐T2D) to maintain normal body weight (BW) and glycemia until 40 weeks of age. Hyperphagic O‐T2D rats had significantly greater BW, body fat, and blood glucose than O‐CON. Total cross‐sectional area (Tt.Ar), cortical area (Ct.Ar), Ct.Ar/Tt.Ar, and polar moment of inertia of the mid‐diaphyseal femur adjusted for BW were greater in O‐T2D rats versus O‐CON. Whole‐bone biomechanical properties of the femur assessed by torsional loading to failure did not differ between O‐T2D and O‐CON, but tissue‐level strength and stiffness adjusted for BW were reduced in O‐T2D relative to O‐CON. Micro–computed tomography (μCT) of the distal epiphysis showed that O‐T2D rats had reduced percent bone volume, trabecular number, and connectivity density, and greater trabecular spacing compared with O‐CON. Basal tibial blood flow assessed by microsphere infusion was similar in O‐T2D and O‐CON, but the blood flow response to insulin stimulation in both the proximal epiphysis and diaphyseal marrow was lesser in O‐T2D compared to O‐CON. In summary, impaired insulin‐stimulated bone blood flow is associated with deleterious changes in bone trabecular microarchitecture and cortical biomechanical properties in T2D, suggesting that vascular dysfunction might play a causal role in diabetic bone fragility. © 2017 The Authors. *JBMR Plus* Published by Wiley Periodicals, Inc. on behalf of the American Society for Bone and Mineral Research.

## Introduction

The marked and sustained global increase in the prevalence and incidence of type 2 diabetes (T2D) in recent decades is so alarming that some have described this health crisis as a “diabetes pandemic.”^1^ The health and economic costs of diabetes are enormous, due largely to the vascular complications of T2D, which result in cardiovascular disease, neuropathy, retinopathy, and nephropathy.^1^ A recently recognized complication of T2D is increased fracture risk.^2^ Currently, many potential biomechanical and cellular mechanisms for diabetic bone fragility are under investigation.^3^ However, one mechanism that remains largely unexplored to date, is bone vascular dysfunction and impaired bone blood flow.^4^ Given the essential role of bone blood flow in the maintenance of healthy bone^5^ and the established causal role of reduced blood flow in the pathophysiology of other T2D complications,^6^, ^7^ impaired bone vascular function in T2D warrants further investigation.

The skeleton accounts for 10% to 15% of resting cardiac output^5^ and bone blood flow is essential for nutrient delivery, mineral homeostasis, and maintenance of interstitial fluid flow, which is needed for mechanotransduction.^8^ Bone blood flow is also a primary mechanism by which bone resorption and formation are coupled during remodeling.^9^ Human and animal data show that bone loss is associated with reduced bone blood flow.^10^, ^11^ Cross‐sectional and prospective, longitudinal studies support a positive association between bone blood flow and bone mass.^12^, ^13^ Likewise, both bone blood flow and bone mass decline with age and after menopause.^14^ Femoral blood flow is reduced in Zucker diabetic fatty rats with long‐term diabetes in association with reduced endothelium‐dependent vasodilation and femoral cancellous and cortical bone mineral density (BMD).^15^

Vascular dysfunction is a characteristic feature of T2D. In particular, the hemodynamic response to insulin is impaired in T2D.^16^ This impaired hemodynamic response to insulin has been observed in numerous tissues, including intestine,^16^ brain,^16^ nerve,^17^ and muscle,^18^, ^19^ but, to our knowledge, has not been investigated in bone. In the vascular endothelium, insulin activation of the pI3/Akt pathway^20^ promotes vasodilation mediated by endothelial‐nitric oxide synthase (eNOS) and reduces synthesis of deleterious adhesion molecules (eg, vascular cell adhesion molecule 1 [VCAM‐1]). Thus, the selective insulin resistance of the vascular endothelium that occurs in T2D^21^, ^22^, ^23^ promotes vasoconstriction, adhesion, inflammation, and oxidative stress, which lead to cardiovascular disease, neuropathy, retinopathy, and nephropathy.

Thus, the present study investigated the effects of T2D on insulin‐stimulated bone blood flow and biomechanical properties by comparison of hyperphagic, diabetic Otsuka Long‐Evans Tokushima Fatty (OLETF; model of hyperphagia‐induced obesity resulting in insulin resistance and T2D^24^) rats (O‐T2D) with normoglycemic, normal weight, control OLETF rats (O‐CON). We hypothesized that relative to normoglycemic O‐CON rats, O‐T2D rats would have reduced insulin‐mediated bone blood flow and deteriorated trabecular microarchitecture, cortical geometry, and biomechanical properties.

## Materials and Methods

### Animal protocol and experimental design

We used a longitudinal, parallel study design to test the hypothesis that OLETF‐T2D rats would have reduced insulin‐stimulated bone blood flow and deteriorated trabecular microarchitecture, cortical geometry, and biomechanical properties. The hyperphagic OLETF rat progressively accumulates excess body fat, which leads to insulin resistance and eventually hyperglycemia and T2D after skeletal maturity.^25^ This accurately parallels what occurs in humans who are overweight during adolescence and develop metabolic complications in adulthood.^26^

One set of animals (*n* = 8 per group) was used to evaluate bone blood flow by microsphere infusion and another (*n* = 8 per group) was used to evaluate trabecular microarchitecture, cortical geometry, and biomechanical properties. Two sets of animals were used because of the interference of the microspheres with analysis of the micro–computed tomography (μCT) scans. Four‐week old, male, OLETF rats were supplied by Otsuka Pharmaceutical (Tokushima, Japan). Upon arrival, rats were divided into two groups: *ad libitum* fed (O‐T2D) or 30% calorically restricted (O‐CON) until 40 weeks of age. We have previously shown that 30% caloric restriction prevents obesity and the development of T2D in hyperphagic OLETF rats,^27^ and that O‐CON are protected against the detrimental skeletal effects of T2D.^28^ Animals were housed individually in a temperature‐controlled (21°C) room with a 6:00am–6:00pm light and 6:00pm–06:00am dark cycle maintained throughout the duration of the experiment. All animals were provided standard rodent chow (Formulab 5008; Purina Mills, St. Louis, MO, USA) in clean cages at the beginning of the week. Body mass and food intake were measured weekly throughout the experimental period. At 40 weeks of age, following an overnight fast, animals were anesthetized with 0.1 mg/kg ketamine, underwent a dual‐energy X‐ray absorptiometry scan and a euglycemic hyperinsulinemic clamp, during which tibial blood flow was measured by microsphere infusion. Following the euglycemic hyperinsulinemic clamp, animals were euthanized via cervical dislocation and exsanguination, and blood and tissues collected (see details for each set of animals used for geometry/biomechanical properties vs. those used for blood flow). The animal protocol was approved by the IACUC at the University of Missouri, and was conducted in compliance with the guiding principles in the Guide for Care and Use of Laboratory Animals.^29^

### Metabolic outcomes

Body mass and food intake were measured weekly throughout the experimental period. Fasting blood samples were taken prior to euthanasia, and serum glucose (Sigma, St. Louis, MO, USA) and insulin (Linco Research, St. Charles, MO, USA) levels were measured using commercially available kits, as described.^27^ Whole‐body composition and BMD were measured using a Hologic QDR‐1000/w DXA scanner calibrated for rats and small animals (Hologic, Bedford, MA, USA; software version 3.6). Isolated femurs were scanned on the same machine (Hologic, Bedford, MA, USA; software version 3.6) for bone‐specific BMD.

### Cortical geometry and trabecular microarchitecture

Animals used for assessment of cortical geometry, trabecular microarchitecture, and biomechanical properties were anesthetized and euthanized as described in Animal Protocol and Experimental Design. Blood and hind limbs were removed and femurs were cleaned of all soft tissue, wrapped in PBS‐soaked gauze, and stored at −80°C for subsequent analysis. Right femurs were scanned using μCT (SIEMENS Inveon Micro‐CT scanner; Siemens, Knoxville, TN, USA) at the Biomolecular Imaging Center (Harry S. Truman Memorial VA Hospital, Columbia, MO, USA) in accordance with the guidelines established by Bouxsein and colleagues.^30^ Scans were performed at a 31.6‐μm^3^ voxel size, with a peak X‐ray potential of 80 kVp and 600 μA. Cortical bone was analyzed at the midpoint of the diaphysis, which was determined using the midpoint between the base of the third trochanter and the distal epiphysis. A 1‐mm (500‐μm in each direction from the midpoint) region of the diaphysis was used for analysis. A 1‐mm region of the distal epiphysis was used for analysis, beginning 1 mm below the end of the distal growth plate. Uniform regions of interest and global thresholds for segmentation were used for each sample. Scans were analyzed using BoneJ software,^31^ a subset of ImageJ (NIH, Bethesda, MD, USA; https://imagej.nih.gov/ij/). Trabecular number was calculated from trabecular spacing and thickness (Tb.N = 1/[Tb.Th + Tb.S]).^32^

### Cortical bone biomechanical properties

Whole‐bone and tissue‐level biomechanical properties of the right femur were assessed using torsional loading to failure, as described.^25^ Briefly, the proximal and distal ends of the right femur were affixed in cement to a steel holder, with crossbars at each end. The distal crossbar was fixed, while the proximal crossbar was rotated around its long axis at a speed of 10 mm/s with a 100‐kg load cell. The machine's software (Stable Micro Systems, Surrey, UK) measured cable force (F) and applied torque (T). The load‐displacement curve from this analysis is analogous with the torque‐twist curve, which, along with geometrical data from the analysis of the μCT scans, was used to calculate: maximal torque at fracture (Tmax), torsional stiffness (Ks), shear modulus of elasticity (G), and ultimate tensile strength, or maximal shear stress (Su), as described.^25^

### Insulin‐stimulated bone blood flow

Animals used for the assessment of tibial blood flow were anesthetized and then underwent a 60‐min euglycemic, hyperinsulinemic clamp as described.^33^ Briefly, a catheter (polyethylene‐50 tubing) was inserted into the right jugular vein to facilitate constant insulin (10 mU · kg · min^−1^; 0.400 μIU · mL^−1^; Novolin R, Novonordisk, Plainsboro, NJ) and variable glucose (0.4 g · mL^−1^; EMD Millipore, Darmstadt, Germany) infusions. The glucose infusion rate was adjusted to maintain euglycemia, which was determined by measurement of blood glucose every 5 min for the first 20 min and every 10 min thereafter. A second catheter was inserted into the carotid artery and advanced to the aorta for microsphere infusion. A third catheter was inserted into the caudal artery for withdrawal of the microsphere reference sample and for monitoring mean arterial blood pressure (MAP) by connection of the catheter to a pressure transducer (PX272; Edwards Lifesciences, Irvine, CA, USA).

The radioactive microsphere technique is the gold standard method for the measurement of regional blood flow.^34^ Microspheres are infused into the systemic circulation and become trapped in the tissue of interest during their first pass through the circulation. Consequently, the number of microspheres trapped in the tissue is proportional to tissue blood flow. A nonradioactive alternative is the use of stable isotopic tracers that can be assayed by neutron activation.^35^ Neutrons penetrate the tissue sample and activate the stable isotope to render it temporarily radioactive, which permits quantification using high‐resolution radiation counting equipment to generate counts per minute (CPM). The assumptions of the microsphere infusion technique are satisfied when the technique is applied to determination of bone blood flow.^36^, ^37^ In addition, the method is reproducible and physiologic changes in bone blood flow are detectable.^38^ In the present study, basal and insulin‐stimulated bone blood flow were measured by infusion of two distinct microspheres (15 μM diameter) each labeled with a different stable isotope before and after the euglycemic hyperinsulinemic clamp (^153^samarium and ^198^gold, respectively; BioPal, Inc. Worcester, MA, USA). To measure basal and insulin‐stimulated blood flow, one reference blood sample was taken at baseline (^153^samarium microspheres), and another was taken after the 60‐min euglycemic, hyperinsulinemic clamp (^198^gold microspheres). The reference blood sample withdrawal began at a rate of 0.6 mL · min^−1^ and was followed (∼10 s later) by infusion of a well‐mixed suspension of the respective microspheres and a saline flush (performed over ∼20 s).

Following the euglycemic, hyperinsulinemic clamp, animals were euthanized as described in Animal Protocol and Experimental Design above, and tibias and kidneys were collected. Tibias were separated into four regions (ie, proximal epiphysis, diaphysis, distal epiphysis, and diaphyseal marrow) for blood flow measures, because regional differences in tibial blood flow have been reported.^15^, ^36^ Individual regions of tibias and kidneys were weighed for blood flow calculations. Microsphere content in blood and tissues was measured by neutron activation as counts per minute (CPM) by the microsphere manufacturer (BioPal, Inc. Worcester, MA, USA). Blood flow was calculated as follows: Blood flow (mL · 100 g^−1 ^· min^−1^) = (0.6 mL · min^−1^ × CPM^−1^_RBS_) × (CPM_tissue_ × [tissue weight]^−1^) × 100, where RBS is the reference blood sample. Adequate mixing of microspheres was assured by comparing blood flow in the right and left kidneys. Two animals in the O‐T2D group were excluded from the analysis due to insufficient and/or nonuniform perfusion. MAP was used to calculate the measures of vascular conductance (VC; the quotient of bone blood flow and MAP [flow/MAP]) and vascular resistance (VR; the quotient of MAP and bone blood flow [MAP/flow]).

### Statistics

Independent sample *t* tests were used to determine differences between O‐T2D and O‐CON for body mass, fasting insulin, and glucose concentrations. Body‐size–dependent bone outcomes (ie, BMD, trabecular microarchitecture, cortical geometry, and biomechanical properties) were adjusted for differences in body size using one‐way analysis of covariance (ANCOVA), with final body weight as the covariate.^39^ The effects of insulin‐stimulated blood flow were determined using a two‐factor (INSULIN: pre‐insulin infusion and post‐insulin infusion; and GROUP: O‐T2D and O‐CON) repeated measures ANOVA (RMANOVA). A GROUP × INSULIN interaction with *p* < 0.15 was considered statistically significant to avoid incorrectly concluding that a nonsignificant test for interaction (ie, *p* > 0.05) implies a lack of any interaction; and post hoc *t* tests (pre‐insulin infusion versus post‐insulin infusion within group and O‐T2D versus O‐CON within pre‐insulin infusion and post‐insulin infusion) were used to locate the interaction. One‐tailed *t* tests were used because our hypotheses were directional and *p* ≤ 0.05 was considered statistically significant. Insulin‐stimulated changes in blood flow and vascular conductance are conventionally reported as percent change. Therefore, percent change in blood flow and vascular conductance were also calculated; and group differences in percent changes were evaluated using the Mann‐Whitney *U* test. This secondary statistical analysis was used to corroborate the results of the two‐factor RMANOVA. Data are means ± SD or adjusted means ± SE for ANCOVA results (ie, means adjusted for body weight covariate). All statistics were performed using SPSS 23 software (IBM, Armonk, NY, USA).

## Results

O‐T2D animals had significantly greater body mass (*p* = 0.004) and body fat percentage (*p* = 0.001) than O‐CON (Table 1). As expected, O‐T2D animals had significantly greater blood glucose compared to O‐CON; however, the two groups had similar insulin concentrations. It is well‐established that, at 40 weeks, O‐T2D are hypoinsulinemic due to β‐cell insufficiency,^40^, ^41^, ^42^, ^43^ whereas O‐CON had reduced insulin levels due to energy restriction.^(28)^ In the subset of animals that underwent the hyperinsulinemic, euglycemic clamp, O‐T2D had reduced whole‐body insulin sensitivity compared to O‐CON, as shown by the lower glucose infusion rate (GIR) required to maintain euglycemia (O‐T2D = 9 ± 3 mg · kg^−1^ · min^−1^; O‐CON = 23 ± 3 mg · kg^−1^ · min^−1^; *p* = 0.001). O‐T2D animals had significantly higher total body areal BMD than O‐CON animals (*p* = 0.008); there was no group difference in total femoral BMD (*p* = 0.565).

**Table 1:** Body Composition, Metabolic Markers, and Bone Mineral Density in O‐CON and O‐T2D at 40 Weeks of Age

	O‐CON	O‐T2D
Metabolic outcomes^a^		
Body mass (g)	552 ± 39	614 ± 91^*^
Body fat (%)	18.4 ± 3.1	25.9 ± 9.5^*^
Blood glucose (mM)	11.8 ± 0.6	58.3 ± 18^*^
Insulin (pM)	127.54 ± 33.26	85.55 ± 21.02
Areal bone mineral density^b^		
Total body (g/cm^2^)	0.192 ± 0.002	0.203 ± 0.002^*^
Femur (g/cm^2^)	0.303 ± 0.005	0.308 ± 0.005

^a^ Data are means ± SD; O‐CON (*n* = 8) and O‐T2D (*n* = 8).

^b^ Data are means ± SE adjusted for final body weight as a covariate in the ANCOVA; O‐CON (*n* = 8) and O‐T2D (*n* = 8).

^*^ Significantly different than O‐CON.

### Cortical geometry and trabecular microarchitecture

**Figure 1:**
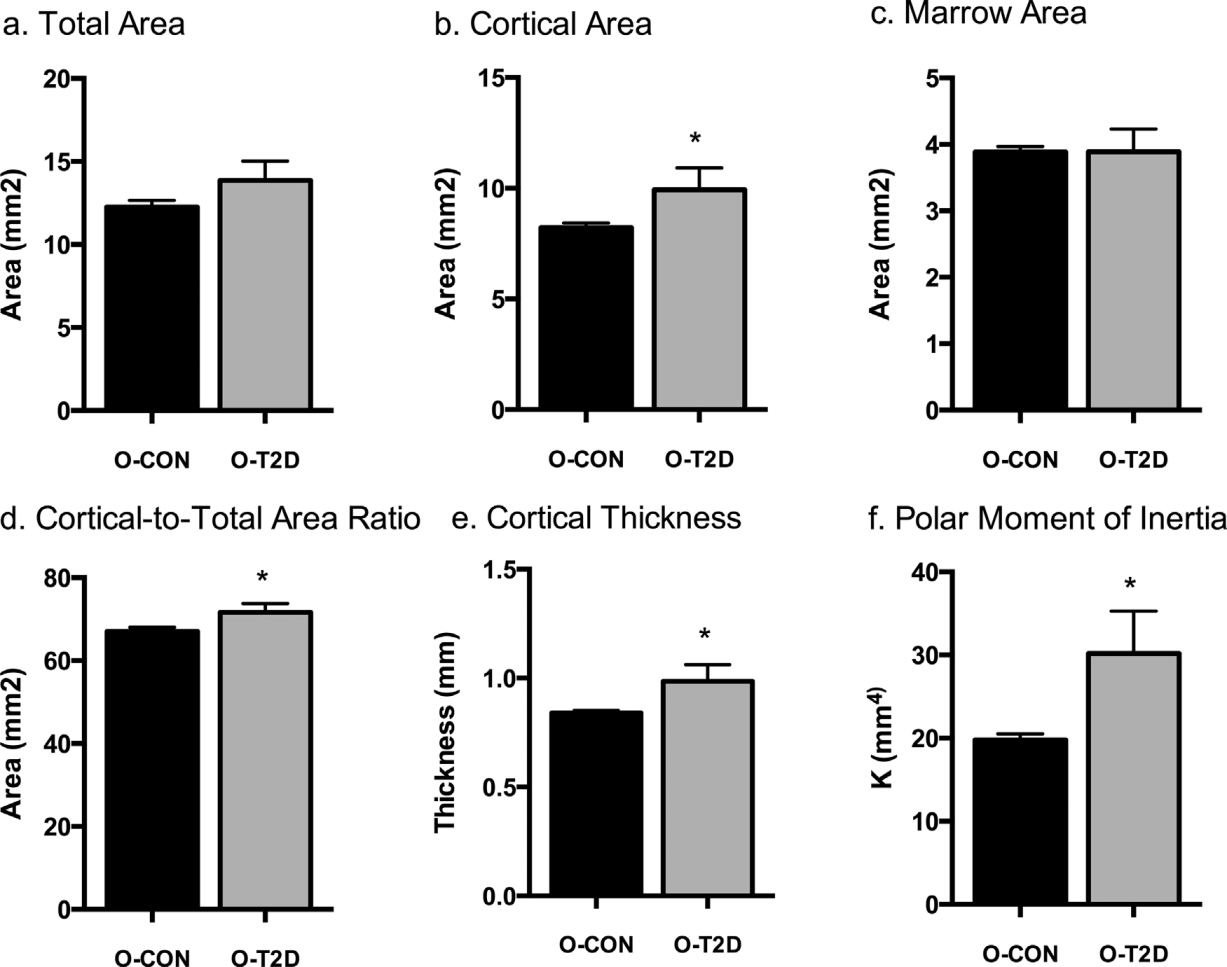
Cortical measurements of the femur: total area (*A*); cortical area (*B*); marrow area (*C*); cortical‐to‐total area (*D*); cortical thickness (*E*); polar moment of inertia (*F*). Data are means ± SE adjusted for body weight; *indicates differences between groups (*p* < 0.05). All groups are 40‐week‐old OLETF rats: O‐T2D (*n* = 8) were allowed *ad libitum* access to a rodent chow diet and O‐CON (*n* = 6) underwent moderate caloric restriction (30% restriction relative to intake of O‐T2D) to maintain normal body weight and glycemia.

**Figure 2:**
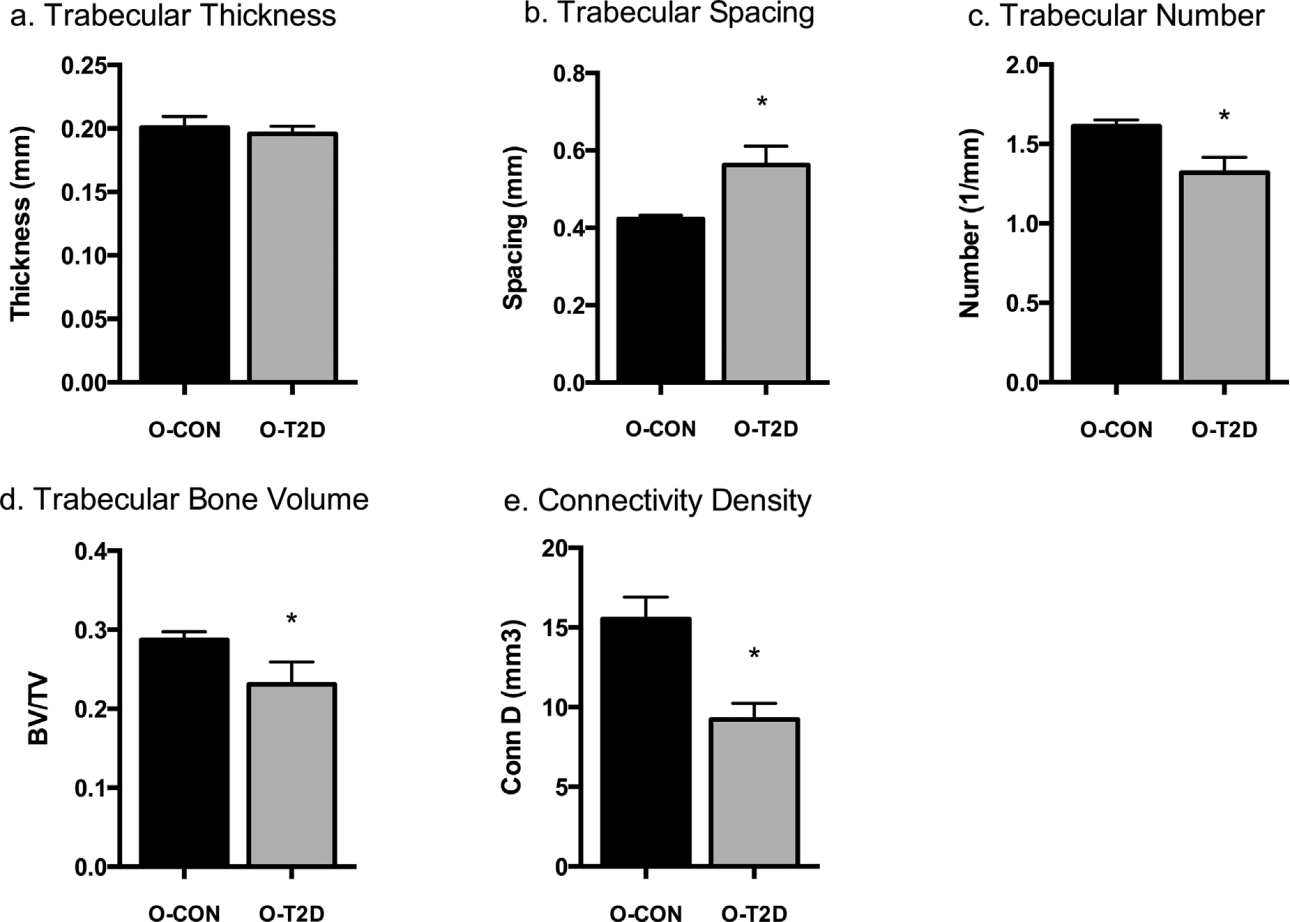
Trabecular measurements of the femur: trabecular thickness (*A*); trabecular spacing (*B*); trabecular number (*C*); trabecular bone volume to total volume (*D*); connectivity density (*E*). Data are means ± SE adjusted for body weight; *indicates differences between groups (*p* < 0.05). All groups are 40‐week‐old OLETF rats: O‐T2D (*n* = 8) were allowed *ad libitum* access to a rodent chow diet and O‐CON (*n* = 6) underwent moderate caloric restriction (30% restriction relative to intake of O‐T2D) to maintain normal body weight and glycemia.

Cortical geometry differed between O‐T2D and O‐CON animals (Fig. 1 *A*–*F*). O‐T2D tended to have greater Tt.Ar (O‐T2D: 13.688 ± 0.347 versus O‐CON: 12.489 ± 0.404, *p* = 0.051; Fig. 1 *A*). Ct.Ar was significantly increased in O‐T2D animals (O‐T2D: 9.814 ± 0.263 versus O‐CON: 8.379 ± 0.307, *p* = 0.005; Fig. 1 *B*), as was Ct.Th (O‐T2D: 0.978 ± 0.021 versus O‐CON: 0.849 ± 0.025, *p* = 0.003; Fig. 1 *E*), polar moment of inertia (O‐T2D: 29.801 ± 1.511 versus O‐CON: 20.335 ± 1.801, *p* = 0.003; Fig. 1 *F*), and Ct.Ar/Tt.Ar (O‐T2D: 28.209 ± 0.640 versus O‐CON: 31.646 ± 0.745, *p* = 0.006; Fig. 1 *D*). There were no differences in Ma.Ar (O‐T2D: 3.852 ± 0.097 versus O‐CON: 3.938 ± 0.113; Fig. 1 *C*). O‐T2D animals had significantly lower BV/TV (O‐T2D: 0.232 ± 0.016 versus O‐CON: 0.287 ± 0.012, *p* = 0.039; Fig. 2 *D*). Tb.Th was similar in both groups (O‐T2D: 0.196 ± 0.010 versus O‐CON: 0.200 ± 0.008, Fig. 2 *A*); however, Tb.Sp was significantly increased (O‐T2D: 0.568 ± 0.012 versus O‐CON: 0.420 ± 0.010, *p* = 0.001; Fig. 2 *B*) and Tb.N was significantly decreased (O‐T2D: 1.313 ± 0.048 versus O‐CON: 1.614 ± 0.037, *p* = 0.004; Fig. 2 *C*) in O‐T2D animals compared to O‐CON. Conn.D was significantly reduced (O‐T2D: 9.256 ± 1.653 versus O‐CON: 15.498 ± 1.276, *p* = 0.031; Fig. 2 *E*) in O‐T2D animals relative to O‐CON.

### Cortical bone biomechanical properties

**Figure 3:**
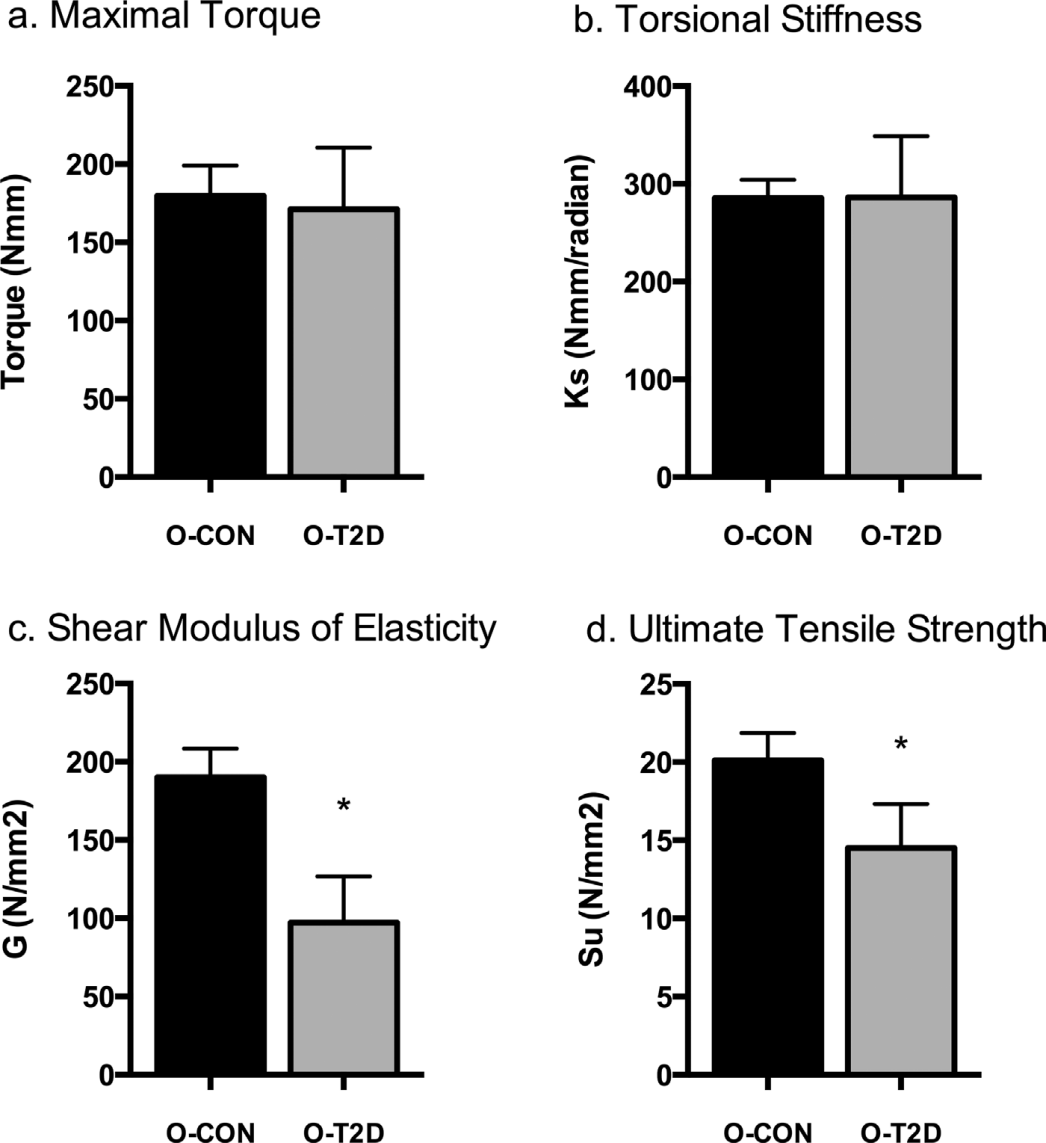
Biomechanical properties of the femur: maximal torque (*A*); torsional stiffness (*B*); shear modulus of elasticity (*C*); ultimate tensile strength (*D*). Data are means ± SE adjusted for body weight; *indicates differences between groups (*p* < 0.05). All groups are 40‐week‐old OLETF rats: O‐T2D (*n* = 8) were allowed *ad libitum* access to a rodent chow diet and O‐CON (*n* = 6) underwent moderate caloric restriction (30% restriction relative to intake of O‐T2D) to maintain normal body weight and glycemia.

O‐T2D and O‐CON did not differ in whole‐bone strength (maximal torque at fracture, Tmax, O‐T2D: 168.970 ± 16.277 versus O‐CON: 182.645 ± 19.405) or torsional stiffness (K, O‐T2D: 280.725 ± 20.503 versus O‐CON: 293.146 ± 24.443) (Fig. 3 *A*, *B*). However, tissue‐level stiffness (shear modulus of elasticity, G) and strength (ultimate tensile strength, Su) were both significantly decreased in O‐T2D relative to O‐CON (O‐T2D: 100.138 ± 13.114 versus O‐CON: 1985.974 ± 15.635, *p* = 0.003 and O‐T2D: 14.339 ± 1.304 versus O‐CON: 20.364 ± 1.555, *p* = 0.017, respectively; Fig. 3 *C*, *D*).

### Insulin‐stimulated bone blood flow

**Figure 4:**
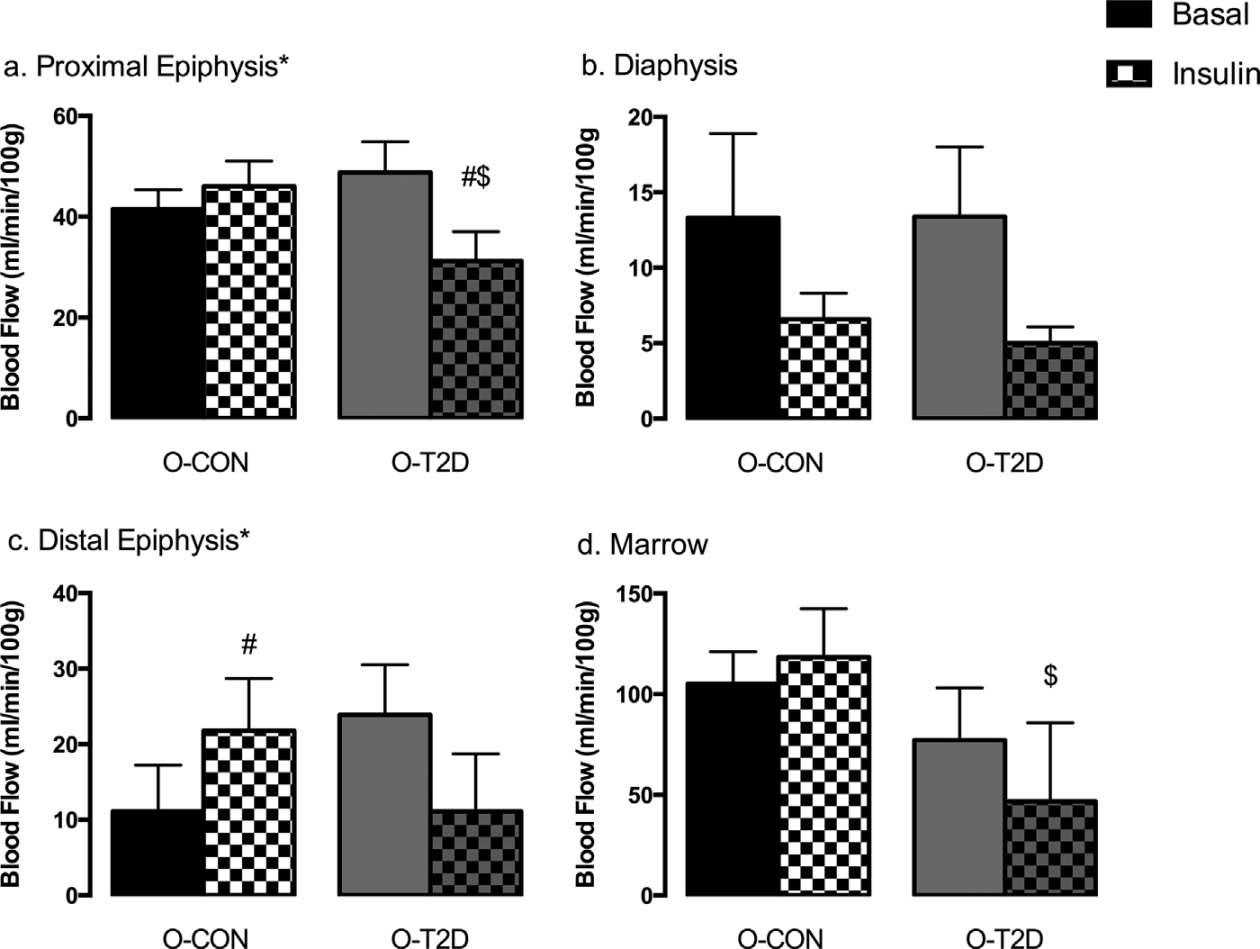
Blood flow measurements of the tibia: proximal epiphysis (*A*); diaphysis (*B*); distal epiphysis (*C*); diaphyseal marrow (*D*). Data are means ± SE; *significant interaction (*p* ≤ 0.15) between group and insulin‐stimulation in repeated measures ANOVA; #significantly different from basal within group (*p* ≤ 0.05, one‐tailed paired *t* test); $significantly different from O‐CON after insulin stimulation (*p* ≤ 0.05, one‐tailed independent sample *t* test). All groups are 40‐week‐old OLETF rats: O‐T2D (*n* = 6) were allowed *ad libitum* access to a rodent chow diet and O‐CON (*n* = 8) underwent moderate caloric restriction (30% restriction relative to intake of O‐T2D) to maintain normal body weight and glycemia.

We observed regional differences in bone blood flow: diaphyseal marrow had the highest blood flow, followed by the proximal and distal epiphyses (Fig. 4 *A*, *C*; Table 2); blood flow was lowest in the diaphysis (Fig. 4 *B*; Table 2). These regional differences are consistent with those previously reported in normal^38^, ^44^ and T2D animals.^15^ Basal blood flow (Fig. 4 *A*–*D*), VC and VR (Table 2) did not differ between O‐T2D and O‐CON.

**Table 2:** Regional Tibial Blood Flow, Vascular Conductance, and Vascular Resistance in Normoglycemic CON and T2D OLETF Rats

	CON		T2D		Two‐factor RMANOVA		
	Basal	Insulin	Basal	Insulin	Group	Insulin	Group × Insulin
MAP (mmHg)	98.6 ± 7.7	82.2 ± 15.2	85.3 ± 10.7	67.0 ± 7.8	0.002	0.003	0.841
Proximal epiphysis							
VC (mL/min/100 g/mmHg)	0.408 ± 0.010	0.582 ± 0.228^*^	0.556 ± 0.211	0.463 ± 0.094	0.821	0.569	0.076
VR (mL/min/100 g)^−1^	2.63 ± 0.83	2.13 ± 1.36	2.15 ± 1.18	2.24 ± 0.48	0.607	0.652	0.511
BF	39.97 ± 9.37	46.01 ± 17.27	48.84 ± 20.95	31.17 ± 7.95	0.643	0.256	0.032
Diaphysis							
VC (mL/min/100 g/mmHg)	0.143 ± 0.078	0.077 ± 0.024	0.149 ± 0.071	0.071 ± 0.027	0.614	0.086	0.446
VR (mL/min/100 g/mmHg)^−1^	8.24 ± 4.50	30.39 ± 18.24	7.60 ± 3.66	11.17 ± 3.17	0.359	0.118	0.196
BF	13.35 ± 7.99	6.56 ± 4.63	13.40 ± 6.46	4.99 ± 2.38	0.759	0.099	0.625
Distal epiphysis							
VC (mL/min/100 g/mmHg)	0.118 ± 0.147	0.257 ± 0.259	0.256 ± 0.178	0.217 ± 0.119	0.294	0.751	0.218
VR (mL/min/100 g/mmHg)^−1^	27.24 ± 30.65	7.43 ± 6.45^*^	4.00 ± 2.25	5.60 ± 2.39	0.112	0.234	0.167
BF	11.31 ± 13.84	19.97 ± 19.38	19.94 ± 17.31	11.39 ± 3.83	0.879	0.879	0.124
Diaphyseal marrow							
VC (mL/min/100 g/mmHg)	1.062 ± 0.358	1.488 ± 0.882^*^	0.833 ± 0.615	0.743 ± 0.351^**^	0.208	0.505	0.314
VR (mL/min/100 g/mmHg)^−1^	1.039 ± 0.348	1.159 ± 1.351	1.614 ± 0.844	1.660 ± 1.014	0.305	0.853	0.934
BF	105.21 ± 38.43	118.39 ± 76.15	77.23 ± 62.94	34.33 ± 21.17	0.164	0.689	0.324

Data are means ± SD; O‐CON (*n* = 8) and O‐T2D (*n* = 6).

VC = vascular conductance; VR = vascular resistance; BF = blood flow.

^*^ Significantly different from basal within group (*p* ≤ 0.05, one‐tailed paired *t* test).

^**^ Significantly different from CON after insulin stimulation (*p* ≤ 0.05), one‐tailed independent sample *t* test.

**Figure 5:**
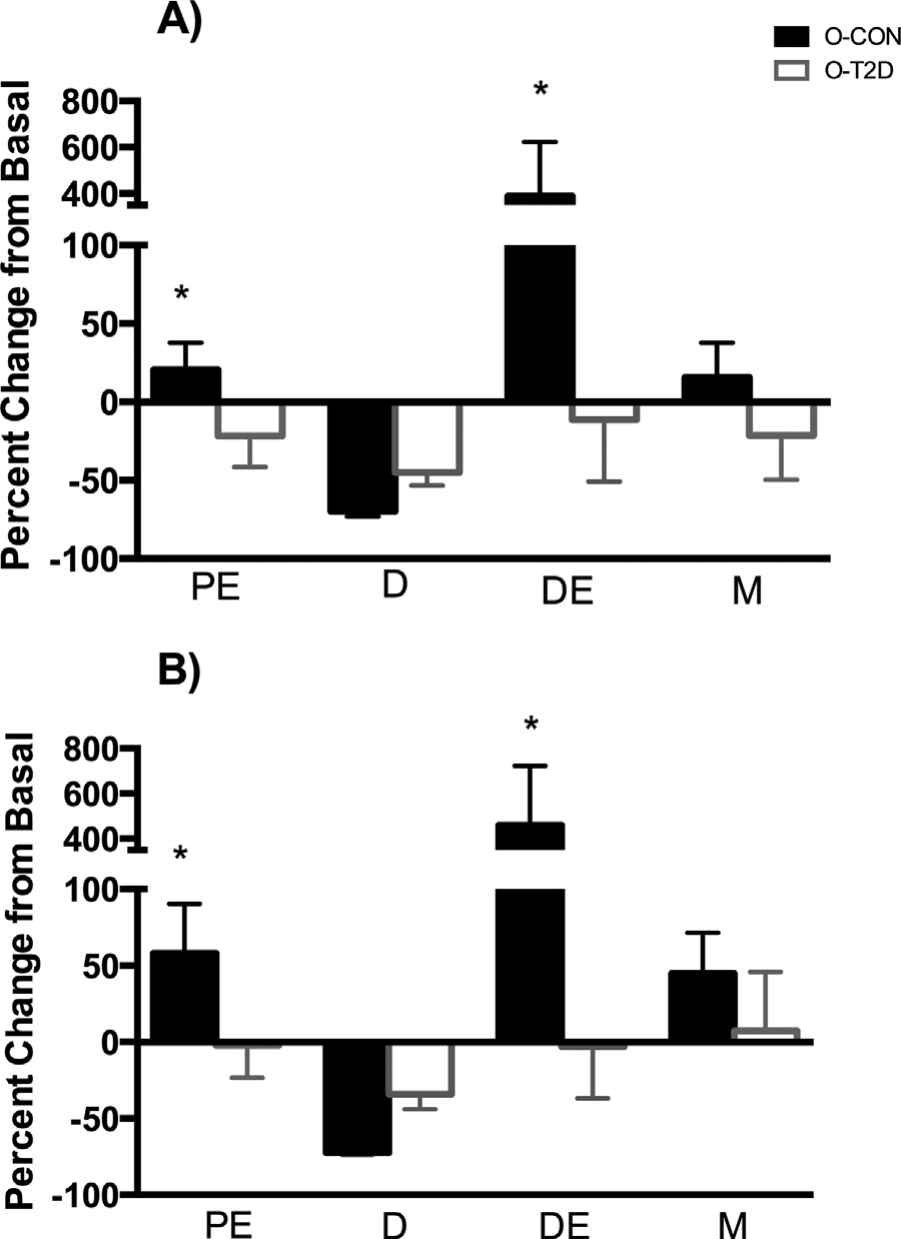
Percent change in blood flow (*A*) and vascular conductance (*B*) in regions of tibia. Data are means ± SE; *significantly different from O‐CON. All groups are 40‐week‐old OLETF rats: O‐T2D (*n* = 6) were allowed *ad libitum* access to a rodent chow diet and O‐CON (*n* = 8) underwent moderate caloric restriction (30% restriction relative to intake of O‐T2D) to maintain normal body weight and glycemia. PE = proximal epiphysis; D = diaphysis; DE = distal epiphysis; M = marrow.

However, O‐T2D and O‐CON showed differential blood flow responses to insulin stimulation in the proximal and distal epiphyses; ie, there were significant interactions in the two‐factor RMANOVA (GROUP × INSULIN interaction *p* values were 0.032 and 0.124, respectively; Fig. 4 *A*, *C*; Table 2). In support of our hypothesis, in O‐T2D blood flow in the proximal epiphysis decreased after insulin‐stimulation (post hoc paired *t* test within O‐T2D *p* = 0.032), such that blood flow after insulin infusion was significantly lower in O‐T2D compared to O‐CON (post hoc *t* test *p* = 0.038). In the distal epiphysis, insulin stimulation increased blood flow in O‐CON (post hoc paired *t* test within O‐CON *p* = 0.049), but not in O‐T2D. In the diaphyseal marrow, insulin‐stimulated blood flow was lower in O‐T2D compared to O‐CON (*p* = 0.016; Fig. 4 *D*). Percent changes in tibial blood flow in response to insulin (Fig. 5 *A*) were consistent with the results of the two‐factor RMANOVA. O‐CON exhibited a greater increase in blood flow in the proximal epiphysis (*p* = 0.046), distal epiphysis (*p* = 0.050), and diaphyseal marrow (*p* = 0.153) compared to O‐T2D.

Changes in blood flow in response to insulin and group differences to the insulin response could be due to changes in MAP and/or VC. There were significant group main effects for MAP (Table 2), such that MAP was greater in O‐CON compared to O‐T2D (O‐T2D: 76.2 ± 2.7 mmHg; O‐CON: 90.4 ± 2.3 mmHg; main effects *p* = 0.002). There was also a significant main effect for insulin stimulation, such that MAP was reduced after insulin stimulation (basal: 92.0 ± 2.5 mmHg; insulin: 74.6 ± 3.4 mmHg; main effect *p* = 0.003). The GROUP‐by‐INSULIN interaction was not significant. Likewise, there was no significant group difference in percent change in MAP after insulin stimulation. However, the percent increases in VC in the proximal and distal epiphyses were greater in O‐CON than O‐T2D (*p* = 0.06 and *p* = 0.05, respectively; Fig. 5 *B*). Thus, greater insulin‐stimulated increases in VC in O‐CON likely accounted for augmented blood flow relative to O‐T2D.

## Discussion

Here, we report for the first time that insulin‐stimulated *bone* blood flow is compromised in the obese, T2D condition, which also adversely affected bone structural and biomechanical properties. We observed compromised insulin‐stimulated vasodilation and blood flow in the proximal and distal epiphyses and in the diaphyseal marrow of T2D animals that was associated with increased vascular conductance. Consistent with our observation, Stabley and colleagues^15^ reported reduced nitric oxide–mediated, endothelium‐dependent vasodilation in isolated femoral principal nutrient arteries in Zucker diabetic fatty (ZDF) rats with a corresponding reduction in bone blood flow. These results suggest that the vascular endothelium of bone, like that of other tissues,^45^ becomes insulin resistant in T2D. Additional in vitro experiments with isolated bone blood vessels are needed to definitively establish that this occurs. Stabley and colleagues^(15)^ also reported reduced basal blood flow in the femur of alert ZDF rats with long‐term diabetes compared with nondiabetic animals. In the present study, we did not observe differences in tibia basal blood flow between anesthetized O‐CON and O‐T2D rats. This result is expected because anesthesia suppresses sympathetic nervous system activity, which increases vasodilation and blood flow. However, the discrepant effects of T2D on basal bone blood flow in our study versus those reported by Stabley and colleagues^15^ might also be due to differences in the animal models of T2D. The ZDF rat has altered leptin signaling because of a missense mutation of leptin receptor, and leptin causes vasorelaxation; therefore, animals with reduced leptin signaling might have reduced basal blood flow.^46^

The results of the present study confirm our previous findings^25^, ^28^ and those of others^2^, ^47^, ^48^ that insulin resistance and T2D associated with excess adiposity negatively impact bone health. Specifically, in the present study, obesity‐induced T2D negatively influenced trabecular microarchitecture and tissue‐level cortical strength and stiffness. Trabecular bone volume was decreased with greater trabecular separation and reduced connectivity density in O‐T2D rats relative to O‐CONs. Although T2D reduced tissue‐level strength and stiffness of the femur, whole‐bone biomechanical properties were not compromised, most likely due to compensatory changes in bone geometry, such as increased cortical area and thickness. The effects of T2D on bone health observed in the hyperphagic, obese OLETF rat are consistent with impaired bone microarchitecture and biomechanical properties that occur in obese/diabetic humans.^49^, ^50^

Given the important hemodynamic role of insulin in nutrient delivery, the current data provide novel insight regarding how impaired insulin‐stimulated bone blood flow contributes to increased bone fragility. Although the effects of insulin on glucose uptake by skeletal muscle and adipose tissue are well characterized, how insulin affects glucose uptake by bone is not thoroughly understood. Some studies suggest that insulin signaling does not alter expression of glucose transporters.^51^, ^52^ However, a recent study of primary osteoblasts showed that GLUT4 expression increases with osteoblast differentiate into mature osteoblasts, and insulin exposure increases GLUT4 expression that is necessary for insulin‐stimulated glucose uptake.^53^ By contrast, basal glucose uptake appears mediated by insulin‐independent GLUT1.^51^ Thus, it appears that insulin increases glucose uptake in mature osteoblasts via increased osteoblast expression of GLUT4. Regardless of the direct effects of insulin on glucose uptake via alterations in glucose transporter expression, increased bone blood flow in response to postprandial insulin release increases delivery of oxygen, nutrients, growth factors, and hormones. In particular, delivery of circulating growth factors and hormones, the release of which is upregulated in response to feeding, is augmented as a result of insulin‐stimulated blood flow. Thus, impaired insulin‐stimulated bone blood flow might adversely affect bone due to reduced delivery of nutrients and other compounds that are essential for normal bone.

In particular, reduced postprandial availability of insulin and glucose has the potential to have significant deleterious skeletal effects because of the critical roles of insulin and glucose in osteoblast and osteoclast development and function. Insulin stimulates osteoblastogenesis and osteoblast differentiation,^54^ inhibits osteoblast apoptosis,^51^, ^55^ and reduces osteoclastogenesis.^52^ Osteoblast‐specific deletion of the insulin receptor impairs bone acquisition in mice, with up to 50% decreases in trabecular bone.^56^ Reduced glucose uptake in bone causes decreased bone formation and reduced bone mass in GLUT1 knockout mice.^51^ Thus, although the direct effects of insulin on glucose uptake in bone are not entirely clear, insulin has other essential direct actions on osteoblasts and osteoclasts.^57^

In the present study, insulin‐stimulation increased bone blood flow despite a decrease in MAP in the normoglycemic O‐CON rats. Because blood flow is the product of MAP and VC, the increase in bone blood flow observed in the O‐CON control animals was due to increased VC, ie, vasodilation, in response to insulin. By contrast, insulin did not increase blood flow or VC in the O‐T2D rats. These findings as well as the reduced NO‐mediated vasodilation in femoral primary nutrient arteries isolated from ZDF rats^58^ suggest eNOS production or availability is reduced in T2D. In a study of cerebral vascular function in the animals used in the present study to assess tibial blood flow, NOS‐dependent vasodilation was reduced, whereas endothelin‐1 (ET‐1)‐mediated vasoconstriction was increased in isolated cerebral arteries of diabetic O‐T2D animals.^59^ These observations are consistent with our hypothesis that the T2D‐associated reduction in insulin‐stimulated bone blood flow is due to altered insulin vasodilatory response in the vascular endothelium of T2D animals. Although the response of the bone vascular endothelium to insulin appears altered in T2D, the responsiveness of the bone vascular endothelium to other physiologic stimuli, such as exercise or hormones, known to cause vasodilation and increased blood flow in T2D, has not been studied to date.

Moreover, other pathogenic processes associated with endothelial dysfunction might negatively impact bone in T2D. For example, increased inflammation,^60^ cellular adhesion,^61^ and oxidative stress^62^ might also contribute to the changes in bone structural and material properties that occur in T2D. Impaired bone endothelial function is just one potential mechanism by which T2D results in bone fragility. Glucotoxicity,^63^ lipotoxicity,^52^ and reduced Wnt/β‐catenin signaling^64^ also contribute to diabetic bone fragility by compromising the osteogenic potential of mesenchymal stem cells,^65^ reducing osteoblastogenesis, increasing osteoblast apoptosis, impairing osteoblast function, and increasing osteoclast number and activity.^66^ Moreover, accumulation of advanced glycation end‐products (AGEs) negatively affect bone's material properties,^67^ impairs osteoblast differentiation,^68^ and mineralization,^69^ and increase sclerostin expression and apoptosis in osteocyte‐like cells.^70^ Thus, dysfunction of bone endothelial cells is just one of many interrelated mechanisms of diabetic bone fragility.

The present study has potential limitations that are inherent to investigation of the skeletal effects of obesity/T2D. First is the confounding effect of differences in body mass/weight gain among treatment groups on body‐mass–dependent bone outcomes. We addressed these issues by including body mass as a covariate in the statistical analyses for body‐size–dependent bone outcome variables (ie, cortical geometry and biomechanical properties) as recommended by Jepsen and colleagues.^39^ Similarly, obesity might affect blood flow through mechanisms unrelated to insulin resistance and T2D. Although the hyperphagic OLETF rat model of T2D does not allow us to disentangle the effects of excess body mass (adiposity) from the metabolic and endocrine/paracrine changes associated with obesity, the model replicates human T2D.

In summary, the present study assessed insulin‐stimulated bone blood flow using stable‐isotope–labeled microspheres. We observed compromised insulin‐stimulated bone blood flow, which was associated with deleterious changes in bone trabecular microarchitecture and cortical biomechanical properties in T2D OLETF rats. The results of this study suggest that vascular dysfunction might contribute to the pathogenesis of diabetic bone fragility in addition to other mechanisms. To investigate this hypothesis, future studies are warranted that examine whether changes in insulin‐stimulated blood flow precede changes in bone structure and biomechanical properties, as well as osteoblast and osteoclast differentiation and activity during the pathogenesis of obesity‐associated T2D.

## Disclosures

All authors state that they have no conflicts of interest.
